# Current State of Radiolabeled Heterobivalent Peptidic Ligands in Tumor Imaging and Therapy

**DOI:** 10.3390/ph13080173

**Published:** 2020-07-30

**Authors:** Benedikt Judmann, Diana Braun, Björn Wängler, Ralf Schirrmacher, Gert Fricker, Carmen Wängler

**Affiliations:** 1 Biomedical Chemistry, Department of Clinical Radiology and Nuclear Medicine, Medical Faculty Mannheim of Heidelberg University, 68167 Mannheim, Germany; Benedikt.Judmann@medma.uni-heidelberg.de (B.J.); Diana.Braun@medma.uni-heidelberg.de (D.B.); 2 Molecular Imaging and Radiochemistry, Department of Clinical Radiology and Nuclear Medicine, Medical Faculty Mannheim of Heidelberg University, 68167 Mannheim, Germany; Bjoern.Waengler@medma.uni-heidelberg.de; 3 Department of Oncology, Division of Oncological Imaging, University of Alberta, Edmonton, AB T6G 1Z2, Canada; schirrma@ualberta.ca; 4 Institute of Pharmacy and Molecular Biotechnology, University of Heidelberg, 69120 Heidelberg, Germany; gert.fricker@uni-hd.de

**Keywords:** avidity, dual targeting, heterobivalency, imaging, peptides, PET, radiolabeling, receptor binding, SPECT, therapy

## Abstract

Over the past few years, an approach emerged that combines different receptor-specific peptide radioligands able to bind different target structures on tumor cells concomitantly or separately. The reason for the growing interest in this special field of radiopharmaceutical development is rooted in the fact that bispecific peptide heterodimers can exhibit a strongly increased target cell avidity and specificity compared to their corresponding monospecific counterparts by being able to bind to two different target structures that are overexpressed on the cell surface of several malignancies. This increase of avidity is most pronounced in the case of concomitant binding of both peptides to their respective targets but is also observed in cases of heterogeneously expressed receptors within a tumor entity. Furthermore, the application of a radiolabeled heterobivalent agent can solve the ubiquitous problem of limited tumor visualization sensitivity caused by differential receptor expression on different tumor lesions. In this article, the concept of heterobivalent targeting and the general advantages of using radiolabeled bispecific peptidic ligands for tumor imaging or therapy as well as the influence of molecular design and the receptors on the tumor cell surface are explained, and an overview is given of the radiolabeled heterobivalent peptides described thus far.

## 1. Introduction

Whole-body imaging techniques are indispensable tools for the characterization of physiological as well as pathological conditions in daily clinical patient care. In particular, molecular imaging, comprising the nuclear medicine imaging modalities positron emission tomography (PET) and single photon emission computed tomography (SPECT), offers the advantage of tissue characterization on a functional level, thus enabling the detection and characterization of functional changes before morphological alterations can be detected using magnetic resonance imaging (MRI) and computed tomography (CT). The most precise and meaningful information can be obtained by combining functional and morphological imaging modalities (e.g., PET/CT, SPECT/CT, or PET/MRI), representing an important basis for sensitive and specific clinical diagnosis of different diseases.

As functional imaging with PET and SPECT plays such a pivotal role for the detection and characterization of neurologic, cardiologic, and especially oncologic pathologies, the number of radiotracers able to address specific pathologic functional changes has been steadily growing over the last decades. As a result, the research for new radioligands, enabling the visualization target structures with higher sensitivity and specificity continues unabatedly. In general, every bioactive compound accumulating in target cells and tissues can form—when radiolabeled with a suitable β^+^- or γ-emitter—a valuable imaging agent for PET or SPECT. Thus, different compound classes have been used for the development of new imaging agents for PET and SPECT, such as small molecules, peptides, peptide mimetics, RNAs, and antibodies.

The imaging of malignant diseases with high specificity mostly utilizes radiolabeled peptides as imaging agents as this substance class is—besides antibodies—able to bind to cell surface receptors that are overexpressed by the respective malignancy with high affinity and specificity, thus allowing for the accurate discrimination between benign and malignantly transformed tissue. Furthermore, peptides usually exhibit low toxicities and immunogenicities, and are readily synthesized and chemically modified to produce homogeneous products with tailored properties. Moreover, they show—in contrast to also highly target-specific and -affine antibodies—a favorably fast tissue penetration, target accumulation, and non-target tissue clearance and thus highly advantageous pharmacokinetic properties. Thus far, numerous radiolabeled peptide drugs have been developed for both diagnostic imaging of peptide receptor expression or peptide receptor radionuclide therapy (PRRT) [1,2,3,4,5,6,7,8].

Over the last years, a large number of radiolabeled peptides were developed for diagnostic imaging and therapy, including multivalent peptides consisting of more than one copy of the targeting peptide. This peptide homomultimer concept bears several advantages. First, the metabolic stability of peptide multimers is commonly increased compared to the respective monomers. This can be attributed to the usually contained artificial structure elements hampering a degradation by endogenous peptidases and to the steric demand of the constructs, resulting in steric hindrance towards peptidase attacks [9,10,11,12]. This results in a longer bioavailability of the compound and thus increased probability of multimer receptor binding. Second, higher target avidities (avidity is the affinity of a ligand being able to bind with more than one target-affine binding unit) are generally obtained, resulting in tighter target binding, higher tumor uptakes combined with higher tumor-to-background ratios and a prolonged tumor retention [13,14,15,16,17,18].

**Figure 1:**
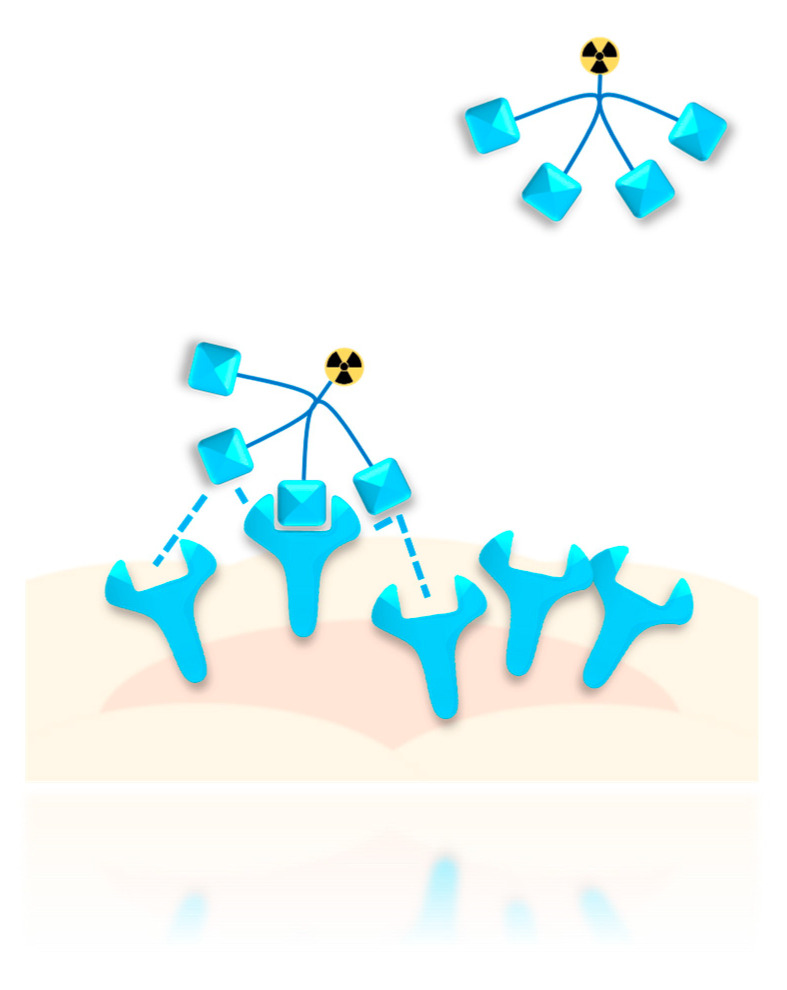
Schematic depiction of the “forced proximity” effect. Considering a peptide multimer having already bound with one peptide to a receptor on the cell surface, unbound peptide copies of the same multimer are located near the cell in close proximity of other free target receptors, increasing the probability of further peptide–receptor interactions or re-binding upon ligand dissociation.

The increased avidity of peptide multimers arises from different factors. One is the possibility of the multimer to concomitantly bind the target cell with more than one peptide copy, resulting in tighter target binding. Such concomitant binding also decreases the possibility of complete ligand dissociation. Even if one interaction is lost, other binding interactions remain and ensure target association. In this case, the effect termed “forced proximity” becomes particularly significant—the unbound peptide copies of the multimer stay near the cell surface due to the one ligand bound to its receptor, forcing the unbound peptides of the multimer to stay in reach of other free target receptors (Figure 1). This increases the probability of unbound peptides of the multimer to interact with other free receptors on the tumor cell surface, while increasing the probability of re-binding in case of ligand dissociation. These effects are particularly pronounced when receptor clustering is triggered by peptide binding [19], further increasing the local concentration of receptors near the site of initial peptide binding, and therefore the probability of further binding events.

Due to these favorable effects of peptide multimerization on target interaction and thus tumor targeting, this approach was adapted to different peptide systems such as RGD-based peptides (RGD = peptide with the sequence Arg-Gly-Asp) targeting integrin α_v_β_3_ [9,10,15,17,20,21,22,23,24,25,26,27,28], bombesin analogs binding the gastrin-releasing peptide receptor (GRPR) [29,30,31,32], somatostatin derivatives binding somatostatin receptors (SSTRs) [33,34,35], analogs of α-melanocyte-stimulating hormone (α-MSH) addressing melanocortin receptors [36,37,38,39], minigastrin analogs binding to the cholecystokinin-2 receptor (CCK2R) [11,12], and neurotensin derivatives targeting neurotensin receptors [40,41,42].

However, a severe limitation for receptor targeting persists, irrespective of the valency of the applied peptide—not every tumor of a certain entity expresses or overexpresses a certain receptor and inter- and intra-individual differences can occur [43,44,45,46], limiting the sensitivity of tumor visualization and therapy response. In addition, the receptor status can change significantly upon disease progression (by tumor de-differentiation and metastasis [44,47,48] or induced by tumor therapy [49,50,51]), further limiting successful tumor targeting. To give an example, human breast cancer cells overexpress the GRPR and the neuropeptide Y receptor subtype 1 (NPY(Y_1_)R) in about 75% and 66–85% of all cases, respectively [52,53]. Thus, a GRPR- or NPY(Y_1_)R-monospecific peptide binder, irrelevant if monovalent or multivalent, can only address a portion of breast cancer lesions. Given the fact that the vast majority of these tumors (93% [54]) overexpresses at least one of them or even both, the direction for the development of highly specific and also sensitive radiolabeled imaging agents becomes clear. Combining two peptides that are able to specifically bind to the GRPR or the NPY(Y_1_)R into one heterobivalent radioligand enables a considerably higher cancer targeting efficiency and sensitivity compared to the respective monospecific radioligands.

This concept is not only promising for human breast cancer but for several cancer types where concomitant receptor targeting is warranted. Of course, the heterobivalent or heteromultivalent agents exhibit the same superior properties as homobivalent or homomultivalent peptides compared to their respective peptide monomers and additionally are able to bind to different targets, thus increasing the overall probability of target binding and visualization.

**Figure 2:**
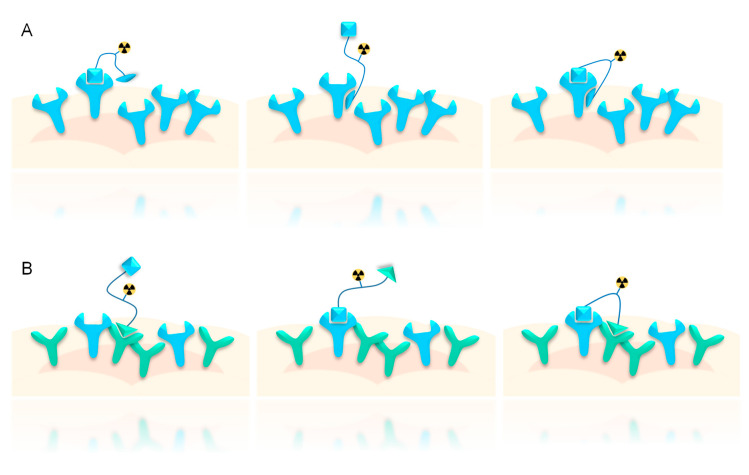
Schematic depiction of bitopic (**A**) vs. heterobivalent (**B**) binding. In the case of bitopic binding, two different binding sites (at least one being allosteric) on the same receptor are addressed by the radioligand, whereas in the case of heterobivalent binding, two orthosteric binding sites of two different receptors are bound.

In general, radiotracers being composed of two different target affine ligands do not necessarily need to address two different structures such as two different receptors (such substances are termed as “heterobivalent” ligands, Figure 2B), but may also contain two different agents addressing the same structure (e.g., one single receptor type) by at least one allosteric interaction. This latter substance class is termed as “bitopic” ligands (Figure 2A).

Although bitopic ligands show tighter target binding compared with monomers and exhibit most of the advantages of homobivalent ligands (vide supra) [55], they cannot take full advantage of the benefits of the heterobivalency approach as they can address only one structure, for example, one tumor-specific receptor, on the target cell (even though it is bound with higher probability and avidity), not resulting in higher sensitivity of target visualization when receptor densities vary between lesions.

Thus, radiolabeled heterobivalent (or heteromultivalent) peptidic agents are most promising for the highly specific and sensitive imaging and therapy of malignant diseases and are thus in the focus of this review. Although only the terms “heterobivalent” or “heterodimeric” will be used in the following review, the same considerations apply to “heteromultivalent” or “heteromultimeric” systems. However, heteromultimers are very rarely described and most of the work was conducted on heterodimeric systems.

### 1.1. General Advantages of Radiolabeled Peptide Heterodimers for Tumor Imaging

As mentioned above, heterobivalent peptidic ligands add several further advantages to those observed for peptide homomultimers (vide supra). In particular, they can bind to different receptors concomitantly or independently overexpressed on the target tissue. This is especially useful in tumor entities or lesions showing no homogenous receptor overexpression between individuals or even in the same patient, which has been shown systematically for several cancer types [54,56,57,58] and should be of general relevance for various other cancers.

Tumors with a non-uniform distribution of target receptors can also be visualized by monospecific peptidic agents but can show an insufficient tumor detection sensitivity due to the partial absence of the target receptor, usually resulting in faint accumulation, or worse, an incomplete detection of all lesions. In principle, it would be possible to address this issue by applying two different monospecific peptidic radioligands. However, this approach has some significant drawbacks. First, the regulatory hurdles were much higher for the examination of a patient using two different radiotracers—if they were injected concurrently, this would result in difficulties regarding dynamic analyses and clinical interpretation of the data as which tracer produced which signal cannot be discriminated. If the tracers were applied consecutively, this would mean two separate examinations, which would be uncomfortable for the patient and furthermore costly. In contrast, a heterobivalent tracer would in contrast have to be applied only once, and even more importantly, some of the intrinsic advantages of heterobivalent ligands such as the higher stability and overall avidity of the combined agents can be taken advantage of.

Furthermore, heterobivalent agents being able to address two different receptor types exhibit a higher overall target binding probability due to the absolute higher number of target receptors and thus a higher tumor visualization rate which is of special importance in cases wherein there is only a moderate receptor expression on the tumor tissue.

### 1.2. Influence of Receptor Density on Heterodimer Binding Mode, Avidity, and Tumor Specificity

**Figure 3:**
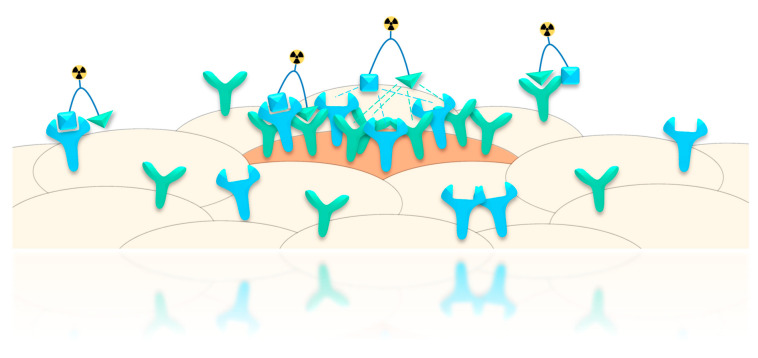
Schematic depiction of increased tumor specificity of heterobivalent binders achieved by concomitant binding to malignant cells overexpressing both receptor types. On benign cells, the target receptors are expressed on a low level, only enabling binding of one of the peptides of the heterodimer, resulting in low target binding being comparable to that of a monospecific peptide. On the malign cell, a higher density of target receptors is available compared to healthy cells, allowing for concomitant binding with higher avidity, resulting in higher targeting specificity.

Another advantage that was proposed for peptide heterodimers—apart from target avidity—is their higher target specificity compared to two monospecific binders. In particular, when two peptides are combined wherein both exhibit a moderate target affinity, a higher tumor specificity can be achieved as benign tissues only express the target receptors in low density whereas the malign cells can express both receptors that can be bound by the heterodimer (Figure 3) [59]. The prerequisite for this increased tumor targeting specificity is thus the expression of both receptors in sufficient number to enable concomitant binding of both peptides of the heterodimer.

**Figure 4:**
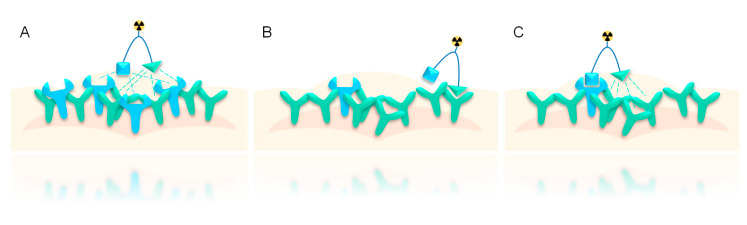
Schematic depiction of possibilities of avidity increase of heterodimeric peptides depending on receptor densities on the target cell. (**A**) Both target receptors are highly overexpressed, resulting in a high density of both receptor types and low distances between the different receptor types. (**B**) and (**C**) Only one of two receptors is strongly expressed, resulting in a high density of only one receptor type while the other is expressed in relatively lower density. In this case, the affinity of that part of the heterodimer is increased, which binds to the receptor expressed in lower density (**C**) while the affinity for the peptide binding to the strongly expressed receptor remains unaltered (**B**).

As mentioned, an important feature of peptide heterodimers is their increased target cell avidity compared to the respective monospecific binders. This increase of avidity is of course most pronounced in cases with a high density of both target receptors on the tumor cell surface, as in such cases, a concomitant binding of both peptides to their target receptors can take place (Figure 4A). This results in tighter binding, increased probability of binding for the second peptide upon binding of the first one, and increased probability of re-binding upon dissociation [18,60].

Even in the case of scarce concomitant binding due to unequal distribution of receptors, with one of them being expressed in lower density than the other, the forced proximity effect nevertheless increases target affinity compared to the respective monomers [18,60]. This overall increase of avidity being usually observed does however not mean that the target affinity is increased for both parts of the heterodimer but can be enhanced for only one of them (Figure 4B,C) [59,61,62,63]. If one receptor type is highly overexpressed on the target cell, resulting in high receptor density, whereas the second receptor is only moderately overexpressed, resulting in a significantly lower receptor density, the peptide heterodimer has two possibilities for receptor binding. (1) It can first bind to the receptor expressed in higher density, resulting in a relatively high distance to the other receptor type and thus not in concomitant binding (Figure 4B). In this case, the affinity for the first peptide binder is not increased. (2) However, if the heterodimer first binds to the less-expressed receptor, the second peptide binder is fixed near its respective receptors, resulting in forced proximity and increased probability of receptor binding for the second binder and thus also an increased probability of re-binding for the initially bound peptide in case of dissociation. This results in an overall increase of avidity for the combined heterodimeric system compared to the peptide monomers (Figure 4C).

Thus, the increase of target avidity of heterobivalent systems is not restricted to such cells expressing both target receptors in high density, but also increased compared to monospecific peptides in the case of tumor cells expressing the target receptors in unequally high density.

### 1.3. Influence of Molecular Design on Target Interaction

**Figure 5:**
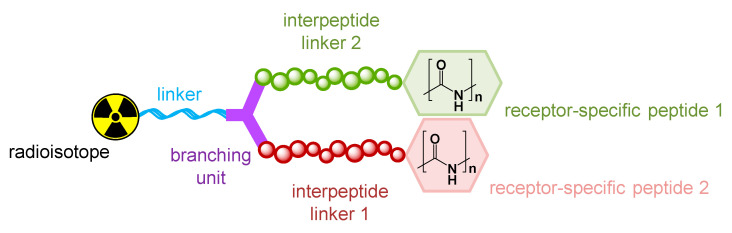
Schematic depiction of the molecular building blocks constituting the radiolabeled heterobivalent peptide.

Heterobivalent peptidic ligands consist of several molecular building blocks: two peptides required for target receptor binding, a branching structure element, a building block for radiolabeling, and linker structures (Figure 5).

For the assembly of peptide heterodimers, it is mandatory that the peptides should be structurally modified as little as possible to preserve their binding affinity, and in particular the peptide’s pharmacophoric sites must not be chemically altered. Furthermore, the branching unit as well as the moiety for radiolabeling are normally somewhat restricted in terms of choice. The branching unit should be relatively small (to keep the molecule relatively small in order to minimize a disturbance of the peptide–receptor interaction due to bulky derivatization) and ideally be symmetrical to avoid the formation of two different (although similar) products. The moiety for radiolabeling is mostly determined by the radionuclide to be used (chelator for radiometal labeling or secondary labeling precursor for introduction of a covalently attached radiolabel).

In contrast to the aforementioned building blocks (peptides, branching unit, and radiolabeling unit), all the linker structures applied can vary significantly and have an important influence on the properties of the resulting heterodimers as they, for instance, determine the distance between both peptidic binders within the molecule. They align the peptides, determine the biocompatibility and in vivo biodistribution characteristics of the resulting heterodimers, and also influence the radiolabeling efficiency of the whole construct.

The linker structure between labeling synthon and the rest of the molecule can, for example, be important to obtain high radiolabeling yields and to positively influence the binding characteristics of the heterobivalent constructs [64,65]. For instance, the labeling reaction using a secondary labeling precursor for ^18^F-introduction was shown to be significantly hampered when the distance between the radiolabeling site and the other parts of the molecule was too small, probably due to steric hindrance of the reaction. However, not only the radiolabeling yields can profit from the introduction of a short linker system in this position, but also the peptide–receptor interactions can be positively influenced, which should be a result of the decreased steric interference of the labeling site with the peptide binders.

The linker structures introduced between the peptides also directly influence the binding properties of the peptides to their respective target receptors. In general, the linkers have to exhibit a sufficient length to enable a certain distance between the peptides and thus their simultaneous binding to both receptors being important to take full advantage of the synergistic effects of peptide heterodimerization. On the other hand, very long linkers increase the entropy of the system and decrease the local concentration of the second binder in terms of monovalent binding and thus the forced proximity effect, resulting in a decreased avidity of the whole heterodimer, limiting its tumor accumulation. This important effect related to the linker length between peptide binders was shown for several peptide homo- and heterodimers and resulted in drastic differences in tumor cell uptakes and binding avidities of the studied radioligands [30,31,59,61,63,66]. Unfortunately, an appropriate linker length can only be determined experimentally and depends on the tumor model used as the distance between receptors varies with receptor density and thus tumor type. However, as mentioned before, concomitant binding is not the only factor rendering peptide heterodimers superior to their respective monospecific counterparts.

Apart from the length of the interpeptidic linkers, their rigidity could in theory also have an influence on peptide–receptor binding as it was conceivable that a high linker rigidity might spatially orient the peptides relative to each other, fixing the geometry of the whole construct [67]. This could be assumed to have a positive or negative effect on receptor binding of both peptides as the flexibility of the systems is restricted. On the one hand, highly flexible linkers might facilitate binding of the first peptide but decrease the probability of the second one to bind due to a higher entropy of the system, resulting in a decreased forced proximity effect. In this case, a spatial orientation caused by a rigid linker might be favorable for the second peptide to bind. On the other hand, highly rigid linkers could limit the motility of the molecule, impeding the first peptide to bind although the second binder might be forced to stay near the cell surface, increasing its binding probability. However, an effect of linker rigidity on the peptide–receptor interaction of peptide homo- or heterodimers—although having been investigated in some studies—could not be shown thus far [59,62,66,68].

## 2. Radiolabeled Peptidic Heterodimers Developed for Improved Tumor Targeting

Within the last 10 to 15 years, the approach to combine different receptor-specific peptides to one heterodimeric radioligand to improve tumor targeting emerged, yielding several agents with improvable tumor imaging characteristics but also other ones demonstrating high potential [69,70,71,72,73]. Over the last five years, the number of agents considerably increased, showing the growing interest in the field of heterobivalent peptides for tumor targeting. Not only the pure number of heterodimers developed grew, but also the receptors that were targeted broadened significantly. At first, the molecular target focus was on GRPR (gastrin-releasing peptide receptor) and integrin α_v_β_3_, with some other agents also targeting SSTRs (somatostatin receptors), MC1R (melanocortin receptor subtype 1), or using the unspecific cell-penetrating peptide TAT (transactivating transcriptional activator) sequence. The potential of the heterobivalency approach beyond these targets was further recognized, resulting in attempts to develop agents addressing PSMA (prostate-specific membrane antigen), NPY(Y_1_)R (neuropeptide Y receptor subtype 1), CCK2 (cholecystokinin-2 receptor), VPAC_1_R (vasoactive intestinal peptide receptor subtype 1), VEFG (vascular endothelial growth factor), and the FRα (folate receptor α). The radiolabeled heterobivalent peptides described thus far are discussed in the following paragraphs and summarized in Table 1. Recently, not only the addressed target receptors broadened significantly, but also the potential application of the agents expanded from mainly PET imaging to PET and SPECT imaging as well as tumor therapy, entailing the use of different diagnostic (^18^F, ^64^Cu, ^68^Ga, ^86^Y, ^99m^Tc, and ^111^In) as well as therapeutic (^90^Y, ^125^I, and ^177^Lu) radionuclides (Table 2).

### 2.1. Heterobivalent Agents Binding the GRPR and PSMA for Improved Prostate Carcinoma Targeting

Although approaches to combine GRPR and PSMA binders to radiolabeled heterobivalent ligands emerged only in 2014, there were several agents already described in this group of heterobivalent peptidic agents, some of them showing high clinical potential.

The reason for developing agents comprising a bombesin-derived agent (targeting the GRPR) and a Glu-urea-Lys or Glu-urea-Glu structure motive (addressing the PSMA) for imaging of prostate carcinoma (PCa) results from the changes of the receptor statuses during progression of the disease. In the early phase of PCa, the GRPR is strongly expressed in high incidence [111]. In contrast, PSMA is highly upregulated during disease progression, metastasis, and de-differentiation [112,113,114,115], but in some cases is missing completely or expressed rather heterogeneously, limiting achievable imaging sensitivities addressing PSMA as the only target [44,116]. Radioligands addressing only one of both target structures are therefore not able to visualize all lesions [117]. Hence, a combined imaging agent binding the GRPR and PSMA would be extremely advantageous for the visualization of malignant tissue within all stages of PCa development.

**Figure 6:**
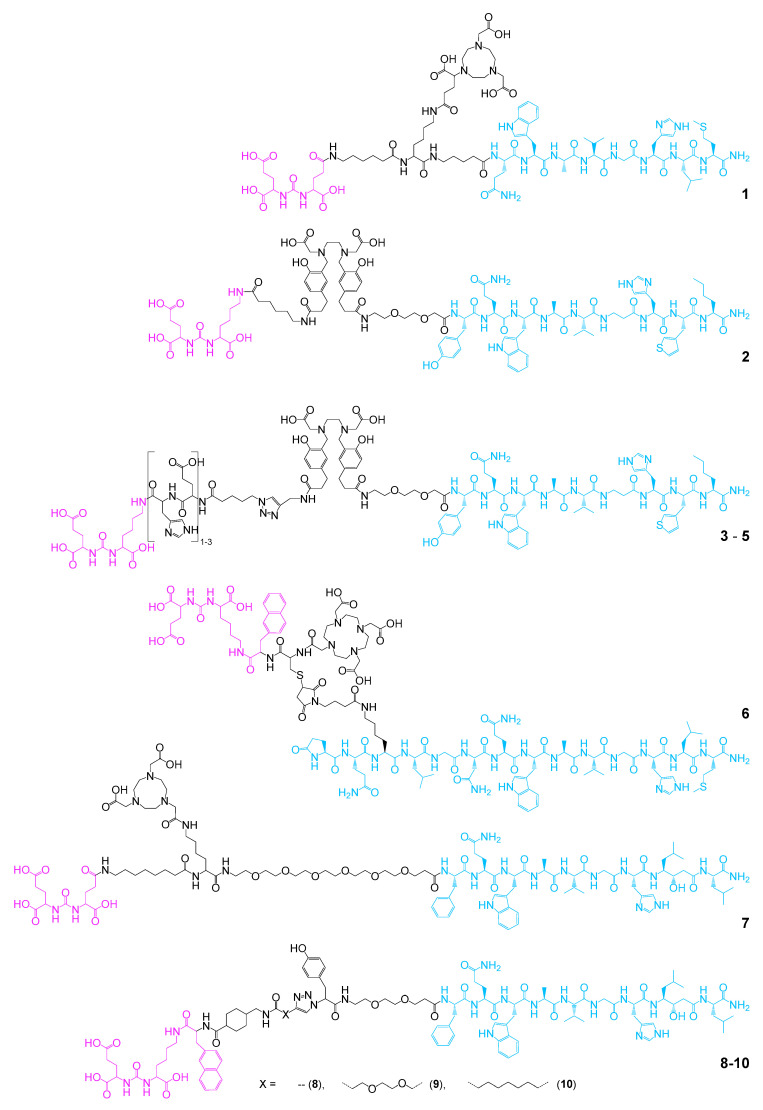
Structures of GRPR- and PSMA-bispecific agents developed for prostate carcinoma (PCa) imaging or therapy. PSMA-specific binders are depicted in magenta, GRPR-specific ones in blue.

In 2014, two examples of heterobivalent GRPR- and PSMA-targeting agents were described. One was composed of the PSMA- and GRPR-binding motives DUPA (Glu-urea-Glu) and BBN_7–14_ (an agonistic truncated analog of endogenous bombesin) modified with the chelator NODAGA ((1,4,7-triazacyclononane-4,7-diyl)diacetic acid-1-glutaric acid) and radiolabeled with ^64^Cu (**1**) [74]. The second was based on Glu-urea-Lys and BZH3 as PSMA- and GRPR-targeting motives, both conjugated to the free carboxylic acid functionalities of the chelator HBED-CC (*N*,*N*′-bis[2-hydroxy-5-(carboxyethyl)benzyl]ethylenediamine-*N*,*N*′-diacetic acid). The resulting bispecific agent was radiolabeled with ^68^Ga (**2**) [75] (Figure 6).

It could be shown for both agents **1** and **2** that they were taken up by GRPR- and PSMA-expressing PC3 and LNCaP tumor cells by specific interaction with the respective target receptors showing good affinities (**1**: IC_50(GRPR)_: 11.1 ± 0.5 nM, IC_50(PSMA)_: 1.2 ± 1.4 nM; **2**: IC_50(GRPR)_: 9.0 ± 1.8 nM, IC_50(PSMA)_: 25.0 ± 5.4 nM) in the same range or slightly decreased compared to the corresponding peptide monomers. The uptake could furthermore be blocked by the respective monomeric agents. These results demonstrated the feasibility of the approach to combine GRPR- and PSMA-targeting agents to one bispecific radiolabeled imaging agent. However, in in vivo experiments in PC3/AR42J and LNCaP tumor-bearing mice, [^68^Ga]Ga-**1** and [^68^Ga]Ga-**2** showed in part massive background uptakes in kidneys, spleen, liver, small intestines, and pancreas, limiting their clinical usefulness. Thus, attempts were made to improve the in vivo biodistribution characteristics of these agents. For this purpose, **2** was modified with one to three His-Glu-units, resulting in compounds **3**–**5** [76] as it was shown before that the introduction of this hydrophilic spacer system can reduce kidney, liver, and spleen uptakes of peptidic radiotracers [118]. Indeed, it could be shown for [^68^Ga]Ga-**4** that the tumor-to-background ratios could be massively improved in xenograft mice. However, the introduction of the linker also reduced the affinities of both parts of the heterodimer to their respective targets by factor 2 to 3. The obtained imaging results were also suboptimal for [^68^Ga]Ga-**4**, still showing a high accumulation in the gastrointestinal tract. Nevertheless, and despite decreased receptor affinities, this work showed that it is possible to considerably enhance in vivo pharmacokinetics of the agents by structural optimization.

In another approach, iPSMA (Glu-urea-Lys-Nal, used as PSMA-targeting agent) was connected to a full-length analog of bombesin (Lys^3^-bombesin for GRPR binding) over a very short cysteine building block carrying a DOTA (1,4,7,10-tetraazacyclododecane-1,4,7,10-tetraacetic acid) chelator to enable radiolabeling not only with the diagnostic radionuclide ^68^Ga, but also offering the possibility to introduce a therapeutic radionuclide to obtain a theranostic pair (**6**) [77,78]. The ^68^Ga-labeled molecule showed [77]—despite the short intramolecular bridge between both target binders—high affinities to both target structures (K_d_s heterodimer: 43.7 ± 3.8 nM for the GRPR and 4.4 ± 2.3 nM for PSMA; K_d_ [^68^Ga]Ga-DOTA-Lys^3^-BBN: 48.8 ± 3.3 nM for the GRPR; K_d_ [^68^Ga]Ga-DOTA-iPSMA: 1.0 ± 0.3 nM for PSMA) and even slightly higher uptakes in GRPR-positive PC3 and PSMA-positive LNCaP cells compared with the respective monomers. These results could be confirmed by in vivo tumor imaging of mice carrying PC3 and LNCaP tumors where the heterodimer visualized the tumors with higher SUV_max_ (SUV = standardized uptake value) than the corresponding radiolabeled monomers.

These results of higher observed tumor uptakes for the heterodimers compared to the respective monomers, although comparable target affinities were obtained for heterodimer and monomers, support the above-mentioned assumption that tumor uptakes can profit from peptide heterodimerization not only in cases of concomitantly high receptor expression but also in cases of the expression of only one target receptor type, nevertheless resulting in higher affinity of the whole heterobivalent construct.

However, no complete biodistribution data were provided in this study, preventing an assessment of achievable tumor-to-background ratios. Interestingly, a non-optimal ^68^Ga-radionuclide incorporation and a relatively low radiochemical yield (RCY) of 62 ± 4% were observed during radiolabeling. In contrast, ^177^Lu-labeling proceeded with high efficiency, giving [^177^Lu]Lu-**6** in high RCY of 99 ± 1% [78]. For [^177^Lu]Lu-**6**, comparably promising in vitro results were obtained compared to [^68^Ga]Ga-**6**. As found for the ^68^Ga-labeled analog, the heterodimer [^177^Lu]Lu-**6** showed a favorable pharmacokinetic profile in mice carrying PC3 and LNCaP tumors and slightly higher uptakes in both tumor types when compared with the respective monomers, confirming the positive results obtained for [^68^Ga]Ga-**6**. Furthermore, the found tumor uptakes were higher in both tumor types than in any other organ at 3 h post injection (p.i.). The organ showing the highest uptake at this time point were the kidneys, reaching about 6.4% ID/g, whereas the PC3 and LNCaP tumors reached about 9.5% and 8.6% ID/g, respectively. At 96 h p.i., a significant washout from the background occurred, further increasing the obtained tumor-to-background ratios. Thus, this agent showed a high potential for dual GRPR- and PSMA-specific targeting, even though the evaluation in only mono-target-expressing PC3 and LNCaP cells is not ideal for the evaluation of the heterobivalent agents in in vitro and in vivo studies as no synergistic effects of heterodimerization on tumor uptakes caused by concomitant binding can be detected.

At the same time, another bispecific agent based on DUPA and RM26 (a GRPR antagonist), being connected by a long and flexible linker structure, carrying a NOTA (1,4,7-triazacyclononane-1,4,7-triacetic acid) chelate (**7**) was developed [79]. Although the resulting heterodimer showed considerably four- to fivefold decreased in vitro target affinities of 4 ± 1 nM and 824 ± 230 nM to GRPR and PSMA, respectively (monomers showed IC_50_ values of 0.41 ± 0.06 nM to GRPR and 160 ± 30 nM to PSMA), the pharmacokinetic profile of the ^111^In- and ^68^Ga-labeled tracers was promising, showing the highest uptake in both cases for the GRPR- and PSMA-positive PC3-PIP tumor (≈12% ID/g for [^111^In]In-**7** and ≈7.5% ID/g for [^68^Ga]Ga-**7**), followed by kidneys (≈10% ID/g for [^111^In]In-**7** and ≈6.5% ID/g for [^68^Ga]Ga-**7**) and pancreas (≈3.5% ID/g for [^111^In]In-**7** and ≈2% ID/g for [^68^Ga]Ga-**7**) at 3 h p.i. Using the dual GRPR- and PSMA-positive PC3-PIP cell line in principle enables investigators to determine synergistic target binding of the heterobivalent radiotracer. Consequently, only a partial blocking effect was observed for tumor uptake of both tracers using the monospecific agents but a complete blocking was demonstrated using a combination of both monomers, showing the heterodimer to be taken up into the tumor by both receptor types. Unfortunately, no direct comparison of tumor uptakes was performed between heterodimer and monomers, not allowing any inference on synergistic effects of heterodimerization.

The same group developed agents **8**–**10** (Figure 6), carrying a tyrosine moiety between both target affine parts of the molecule for ^125^I radiolabeling [80]. Radiolabeling yields varied between 69 and 73% but the molar activities achieved were rather low with ≈0.9 GBq/µmol. In in vitro studies on GRPR- and PSMA-positive PC3-PIP cells, the observed affinities to the GRPR (determined on common PC3 cells) were in a reasonable range of 6–20 nM. In contrast to this, the affinities to PSMA (determined on PC3-PIP cells with blocked GRPR) were comparably lower (80–100 nM). However, no monospecific reference compounds were evaluated under the same conditions, enabling the comparison of the values. All agents furthermore showed a considerable lipophilicity with log*_D_*s of 0.22 (**8**), −0.52 (**9**), and 1.07 (**10**), which resulted in high unspecific background organ uptakes in in vivo studies in PC3 and LNCaP tumor-bearing mice. Furthermore, GRPR-related blocking of the tumor uptake of **8** and **10** in PC3 tumors was not successful, indicating an unspecific tumor uptake mechanism. In contrast, at least a partial blocking of the tumor uptake could be shown for **9**. In LNCaP tumor-bearing animals, the blocking of tumor uptakes was more conclusive (at least for **8** and **9**), showing a PSMA-specific uptake despite low target affinities. In PC3-PIP tumor-bearing mice, high background activities were observed and reasonable tumor-to-background ratios could only be obtained after 24 or 72 h p.i. Thus, a target-specific uptake by both GRPR and PSMA could only be shown for [^125^I]I-**9**, exhibiting the lowest lipophilicity in the line of compounds evaluated.

Thus, although none of the described GRPR- and PSMA-bispecific agents have been studied in a clinical context thus far, some of the developed radiotracers demonstrated high potential for bispecific and therefore highly sensitive PCa imaging during all stages of disease.

### 2.2. Heterobivalent Agents Targeting the GRPR and Integrin α_v_β_3_

**Figure 7:**
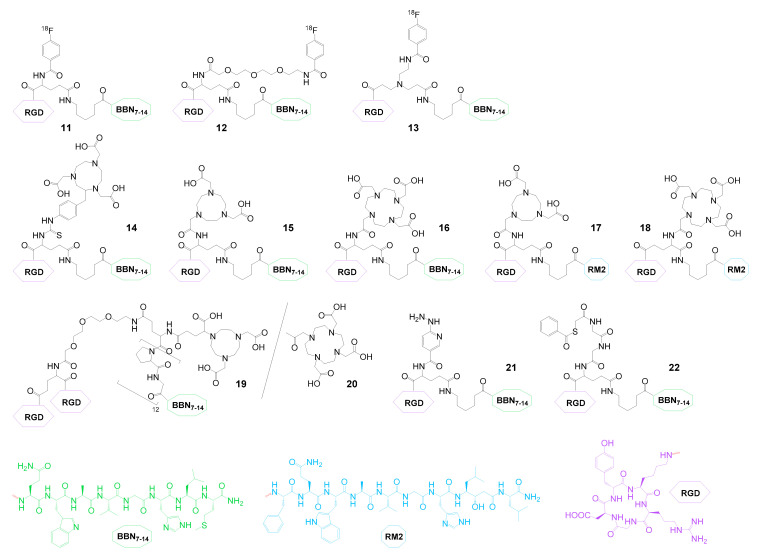
Structures of GRPR- and α_v_β_3_-bispecific agents developed thus far.

The group of radiolabeled heterobivalent peptidic ligands that was developed first and thus far contains the largest number of compounds combines GRPR- as well as integrin α_v_β_3_-specific agents. As mentioned before, the GRPR is overexpressed on a variety of human malignancies, among them prostate, breast, small cell lung, colon, gastrointestinal, neck squamous cell, and pancreatic cancer as well as glioblastomas and neuroblastomas [119]. Integrin α_v_β_3_ is also overexpressed on tumors of different origin, including osteosarcomas; neuroblastomas; glioblastomas; malignant melanomas; and breast, lung, and prostate carcinomas, but more importantly it is an independent marker of tumoral neoangiogenesis, being expressed by endothelial cells of tumoral neovessels [120]. Thus, the combination of GRPR- and α_v_β_3_-specific peptides to radiolabeled heterodimers should be able to increase tumor visualization specificity and sensitivity for the mentioned tumor entities, and several GRPR- and α_v_β_3_-bispecific radiotracers have been developed (Figure 7).

These heterobivalent ligands were radiolabeled with different diagnostic radionuclides such as ^18^F (**11**–**13**), ^68^Ga (**14**), ^64^Cu (**14**–**17**,**19**,**20**), ^86^Y (**18**), ^99m^Tc (**21**,**22**), and ^111^In (**18**), but also with therapeutic nuclides such as ^90^Y (**18**), ^177^Lu (**16**,**18**) and ^188^Re (**22**), mainly intended for imaging and therapy of PCa. However, these agents are not limited to this particular tumor entity. Integrin α_v_β_3_ is overexpressed on invasively growing tumors during metastasis and angiogenesis [121], thus rendering heterobivalent peptide binding to both proteins in principle suitable for efficient synergistic targeting of every GRPR-positive tumor type.

Aside from higher target visualization sensitivity and the common advantages of peptide heterodimerization (vide supra), the combination of BBN (bombesin) derivatives (addressing the GRPR) and cRGD peptides (binding to integrin α_v_β_3_, cRGD = cyclic peptide containing the amino acid sequence Arg-Gly-Asp) brings further favorable effects such as a more balanced in vivo clearance pathway compared to the respective monomers. BBN monomers often show a pronounced hepatobiliary excretion whereas cRGD peptides are usually eliminated relatively fast via the kidneys, resulting in high background uptakes in liver or kidneys, fast clearance, and low tumor uptakes. The combination of both peptides in contrast resulted in a more balanced hydrophilicity/lipophilicity profile combined with lower background accumulation and slower clearance, resulting in higher tumor uptakes [65].

The first described GRPR- and integrin α_v_β_3_-bispecific agents were radiolabeled with ^18^F using *N*-succinimidyl-4-[^18^F]fluorobenzoate ([^18^F]SFB) as a secondary labeling precursor (**11**–**13**). For these agents, an interesting relationship between structural design of the agents and radiolabeling as well as in vitro properties was found—in **11**, where the [^18^F]SFB is directly reacted with the interpeptidic branching unit glutamic acid, relatively low radiolabeling yields of only 12% could be achieved and affinity studies on GRPR-positive PC3 and α_v_β_3_-positive U87MG cells showed somewhat decreased affinities of the heterodimer (IC_50(GRPR)_: 32.0 ± 1.9 nM and IC_50(αvβ3)_: 282 ± 34 nM) compared to the respective monomers (IC_50(GRPR)_: 20.7 ± 3.2 nM and IC_50(αvβ3)_: 202 ± 28 nM) [65]. In addition, **11** showed lower uptakes in in vitro cell uptake studies on PC3 cells than the GRPR-specific monomer, demonstrating no benefit from heterodimerization. A completely different behavior was observed in in vivo studies in PC3 tumor-bearing animals, where [^18^F]**11** displayed higher absolute tumor uptakes than each monomer or a combination of both. Furthermore, higher tumor-to-background ratios as well as a prolonged tumor retention were obtained for the heterodimer compared to the monomers, and it could be demonstrated that its tumor uptake was mediated by both peptide binders. Blocking of GRPR or α_v_β_3_ alone could only partially reduce heterodimer uptake whereas complete blocking was observed by saturating both target structures. These favorable in vitro and in vivo results could be confirmed by the same group studying [^68^Ga]Ga-**14**, [^64^Cu]Cu-**14**, and [^64^Cu]Cu-**16** [83,86] (Figure 7). The aforementioned favorable in vivo properties (being in contrast to the somewhat discouraging in vitro results) can be attributed to the concomitant expression of both target structures on the tumor cell surface under in vivo conditions where the growing tumor obviously additionally expressed α_v_β_3_ instead of GRPR alone. Thus, the full potential of the advantages of peptide heterodimerization (higher avidity, concomitant binding, forced proximity effect, etc.) could be fully exploited here. Another important advantage of the heterodimer is its balanced hydrophilicity/lipophilicity profile (log*_D_* of −0.92 ± 0.04) compared to its respective parent peptides (log*_D_*s of −1.75 ± 0.03 for the ^18^F-FB-cRGD monomer and 1.49 ± 0.02 for the ^18^F-FB-BBN_7–14_ monomer), resulting in decreased uptakes in metabolizing/excreting organs (mainly liver, intestines, and kidneys), further highlighting the positive effects of peptide heterodimerization on in vivo pharmacokinetics.

The same group developed an analog (**12**) of this initial heterodimeric agent, introducing a short PEG_2_ linker between the interpeptidic glutamic acid linkage and the [^18^F]SFB labeling synthon to further improve the properties of the compound [64]. This slight molecular alteration however had significant effects on the chemical, in vitro, and in vivo properties of the heterodimer. It not only resulted in considerably higher radiolabeling yields of 42 instead of 12% (presumably due to reduced steric hindrance of the reactive amino functionality within the interpeptidic branching unit), but also led to higher target affinities (IC_50(GRPR)_: 73.3 ± 1.6 nM and IC_50(αvβ3)_: 13.8 ± 1.8 nM), now being comparable to those of the parent peptide monomers (IC_50(Aca-BBN7-14)_: 79.0 ± 2.1 nM and IC_50(cRGD)_: 11.2 ± 1.4 nM). In vivo, [^18^F]**12** showed slightly higher tumor uptake combined with higher tumor-to-non-tumor ratios than [^18^F]**11**, but also a faster renal clearance, demonstrating the significant impact of small changes in molecular design on the overall properties of the resulting radioligands.

Three radioligands—[^18^F]**12**, [^68^Ga]Ga-**14**, and [^64^Cu]Cu-**14**—were evaluated side by side to identify the most promising agent for potential clinical use [82]. In vitro, **14** and [^19^F]**12** showed comparable GRPR- and integrin α_v_β_3_-targeting abilities but showed considerable differences in their in vivo pharmacokinetic profile. [^68^Ga]Ga-**14** showed the highest tumor uptake and retention in T47D and MDA-MB-435 tumors but also lower tumor-to-background ratios compared to [^18^F]**12** due to slower blood clearance. [^64^Cu]Cu-**14** showed intermediate results and a relatively high liver uptake, indicating a limited stability of the [^64^Cu]Cu–NOTA complex. Two studies conducted by another group, describing the synthesis and partial characterization of [^64^Cu]Cu-**15** and [^64^Cu]Cu-**17** in vitro and in vivo [85,88], found the same, but with an even more pronounced effect of limited stability of the [^64^Cu]Cu–NO2A complex combined with relatively low tumor and high background uptakes. The low stability of the complexes can be attributed to the nature of the used chelate and the insufficient number of free carboxyl functionalities being able to contribute to complex formation [122].

Attempts to further improve the results obtained for **12** by using a symmetrical interpeptidic branching element (**13**) instead of glutamic acid [81] did however not improve cell uptakes and target affinities, shown to be ≈40-fold lower for α_v_β_3_ and ≈2-fold lower for GRPR. These deteriorated binding parameters were also reflected in lower tumor-to-background ratios observed in in vivo studies for [^18^F]**13**. However, from the structural design of the molecule, the reason for these inferior in vitro and in vivo properties of the newly developed agent is not obvious.

Inspired by the positive results that were obtained for some of the ^68^Ga- and ^64^Cu-labeled GRPR- and α_v_β_3_-bispecific ligands, another attempt was recently made to further improve the in vivo pharmacokinetics and tumor uptakes of such agents by combining not only one c(RGDyK) peptide (cyclo(Arg-Gly-Asp-*D*Tyr-Lys)) unit with a GRPR-binding one, but to include two α_v_β_3_-affine peptide copies to obtain NODAGA- and DOTA-modified heterotrimers **19** and **20** [91] instead of heterodimers. The aim was to further increase target interaction and thus in vivo tumor uptakes and retention. An increasing α_v_β_3_ affinity is usually observed for a higher number of RGD peptide units included. However, the applied molecular design unfortunately resulted in a dramatic loss in binding affinity towards the GRPR (IC_50(**19**)_: 24.3 ± 10.9 nM, IC_50(**20**)_: 100.4 ± 73.2 nM, IC_50(BBN)_: 1.36 nM) and also a slightly decreased α_v_β_3_-affinity (IC_50(**19**)_: 165.3 ± 105.4 nM, IC_50(**20**)_: 101.2 ± 57.4 nM, IC_50(RGD2)_: 66.6 nM). Furthermore, the ^64^Cu-labeled heterotrimers failed in in vivo pharmacokinetic studies in a PC3 tumor-bearing mouse model to reproduce or even surpass the positive results obtained before. In contrast, they showed weak tumor uptakes of only ≈1–2% ID/g, which was furthermore not significantly blockable, raising doubts about the receptor-specificity of the observed low tumor uptake. Together with the considerably higher kidney uptakes observed (tumor-to-kidney ratios of approximately 0.2–0.25 after 1 h p.i.), the potential of the developed heterotrimeric agents seems controversial.

Due to its favorable tumor visualization characteristics and the high radiolabeling efficiency, [^68^Ga]Ga-**14** was recently evaluated in 13 patients with histologically confirmed PCa in 2017 [84]. The enrolled patients also received monospecific GRPR-affine ^68^Ga-NOTA-Aca-BBN_7–14_ for direct comparison. The clinical results impressively demonstrated the superiority of the heterobivalent over the monospecific agent, being reflected in higher absolute tumor uptakes (SUV_max_ values for primary tumors, metastatic lymph nodes, and bone lesions of 4.46 ± 0.50, 6.26 ± 2.95, and 4.84 ± 1.57 for [^68^Ga]Ga-**14**, and 2.98 ± 1.24, 4.17 ± 1.89, and 3.61 ± 1.85 for ^68^Ga-NOTA-Aca-BBN_7–14_, respectively) and additionally a higher lesion detection sensitivity of 3 of 4 primary tumors, 14 of 14 metastatic lymph nodes, and 20 of 20 bone lesions, whereas the GRPR-specific tracer was able to identify 2 of 4 primary tumors, 5 of 14 metastatic lymph nodes, and 12 of 20 bone lesions. 

The same agent was in the following also used for clinical breast cancer PET/CT imaging in 22 patients and the results were in part compared to those obtained with the monovalent agent [^68^Ga]Ga-BBN [123]. Also in this case, the heterodimer showed higher SUV_max_ values for primary tumors, metastatic lymph nodes, and bone lesions compared to the monospecific tracer and was able to detect a higher number of lesions ([^68^Ga]Ga-**14**: 13 primary tumors, 8 metastatic lymph nodes, 9 bone metastases and 4 lung metastases; [^68^Ga]Ga-BBN: 11 primary tumors, 3 metastatic lymph nodes, 3 bone metastases and 2 lung metastases). Furthermore, the tumor uptake of [^68^Ga]Ga-**14** correlated well with GRPR and α_v_β_3_ expression, demonstrating the bispecificity of the tracer and the synergistic effects of heterobivalency.

These results clearly substantiate the high potential of bispecific peptidic heterodimers for human tumor imaging.

Besides PET imaging agents, certain GRPR- and α_v_β_3_-bispecifc radiotracers have also been developed for application in SPECT imaging and as agents for endoradiotherapy. One example, [^99m^Tc]Tc-HYNIC(tricine)(TPPTS)-**21** (HYNIC: hydrazinonicotinic acid, TPPTS: triphenylphosphine-3,3′,3″-trisulfonic acid trisodium) [92] showed encouraging in vitro results with only slightly lower affinities of **21** to both target structures than the corresponding monomers. However, in vivo experiments performed with [^99m^Tc]Tc-**21** in a Lewis lung carcinoma mouse model revealed low tumor but high background uptakes due to a fast proteolytic degradation, limiting the usefulness of the tracer. The same group intended to improve the obtained results by changing the used radionuclide chelator system from HYNIC (**21**) to MAG_2_ (2-mercaptoacetylglycylglycyl) (**22**) [93]. For **22**, good in vitro binding affinity data towards the GRPR (IC_50_: 63.3 ± 2.9 nM) and α_v_β_3_ (IC_50_: 13.4 ± 2.5 nM) were found in PC3 and U87MG cells, being comparable to the affinities of the respective monomers Aca-BBN_7–14_ (IC_50_: 71.6 ± 3.1 nM) and c(RGDyK) (IC_50_: 10.8 ± 2.6 nM). In addition, the theranostic potential of the agent was investigated by labeling **22** with ^99m^Tc as well as ^188^Re and evaluation of both radioligands in PC3 tumor-bearing mice. However, a massive accumulation of both agents in background organs (kidneys, liver, spleen, pancreas, intestines)—as already observed for **21**—was found and thus limited the applicability of this theranostic pair.

Two further attempts to obtain a theranostic pair based on a GRPR- and α_v_β_3_-specific agent were described recently by the same group [89,90] and based on precursor **18**, comprising the GRPR antagonist RM2. The utilized DOTA chelator has the advantage of enabling the introduction of different diagnostic as well as therapeutic nuclides, thereby allowing for the investigation of different theranostic pairs. To determine the target affinities in PC3 and U87MG cells, **18** was reacted with the respective cold metal ions ^nat^Y, ^nat^In, and ^nat^Lu to determine a potential influence of the incorporated metal ion, but in all cases, similar IC_50_ values of ≈5.5 nM and ≈350 nM were found for GRPR and integrin α_v_β_3_. Unfortunately, no monospecific peptide references were tested, and thus the affinity values obtained are difficult to put into context. In the following, **18** was labeled with ^111^In (SPECT nuclide) and ^177^Lu (therapeutic nuclide) [90] or ^86^Y (PET nuclide) and ^90^Y (therapeutic nuclide) [89] and evaluated in PC3 tumor-bearing mice. All tracers showed good uptakes into the tumors (7–9% ID/g at 1 h p.i.) which was only completely blockable by a combination of both peptide monomers, demonstrating the tumor uptake to be mediated by both receptor types. Besides uptake into the tumor target, further significant tracer accumulations were observed in the pancreas (7–10.5% ID/g), kidneys (2.4–4.3% ID/g), spleen (0.9–1.4% ID/g), and intestines (1.1–2.7% ID/g) at 1 h p.i. Although the clearance of the tracers was relatively fast from background organs (except kidneys), enabling very good imaging results after 24h, the early high uptake into non-target organs might prevent a therapeutic application of the both therapeutic agents. When directly comparing both theranostic pairs, [^86^Y]Y-**18** and [^90^Y]Y-**18** showed somewhat higher tumor-to-background ratios relative to [^111^In]In-**18** and [^177^Lu]Lu-**18** due to higher tumor and lower non-tumor uptakes, favoring the former combination for further developments.

Interestingly, when directly comparing the GRPR antagonist-comprising [^177^Lu]Lu-**18** and the earlier described GRPR agonist-comprising analog [^177^Lu]Lu-**16** [87], evaluated in PC3-bearing SCID (severe combined immunodeficiency) or immunodeficient nude mice, respectively, [^177^Lu]Lu-**16** showed a significantly less favorable pharmacokinetic profile with much lower tumor-to-non-tumor ratios, especially in pancreas but also in liver, stomach, spleen, kidneys, and intestines. However, the current data cannot explain whether this inconsistency is caused by differences in the animal model or by the often observed more favorable pharmacokinetic profile of GRPR antagonists compared to that of agonists [124].

In the group of heterobivalent peptidic agents targeting the GRPR and integrin α_v_β_3_, several promising radioligands were developed, which showed a high potential for bispecific and therefore highly sensitive imaging of GRPR-expressing tumors. The data reported for the first radiolabeled heterobivalent agent ([^68^Ga]Ga-**14**) that was used in a clinical context for imaging of PCa and breast cancer and was evaluated in direct comparison to the monospecific reference compounds, furthermore clearly substantiated the positive effects of peptide heterodimerization on clinical tumor imaging sensitivity and specificity.

### 2.3. Further Heterobivalent Agents for PCa Imaging or Therapy

Apart from the GRPR- and PSMA- or GRPR- and integrin α_v_β_3_-bispecific heterobivalent agents developed for PCa imaging or therapy, other strategies were followed to improve the targeting of this malignancy using peptide heterodimers.

**Figure 8:**
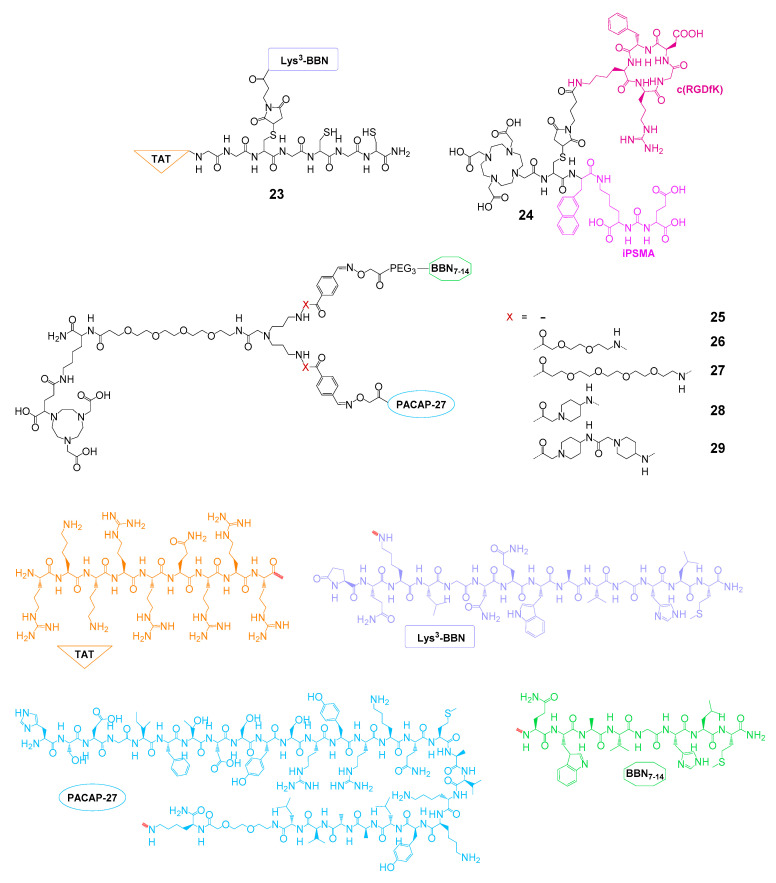
Structures of heterobivalent peptidic agents developed for PCa imaging or therapy, targeting different receptor systems.

One approach aimed at improving SPECT imaging or therapy of PCa by using a ^99m^Tc-labeled heterobivalent agent based on the GRPR-specific Lys^3^-bombesin and TAT_49–57_, serving as a cell-penetrating peptide intended to internalize GRPR-bound dimer into the target cell [94,95] (**23**, Figure 8). The agent was shown to be taken up into PCa PC3 and breast cancer MCF7 and MDA-MB231 cells to a significantly higher extent than the monospecific GRPR-targeting reference peptide (up to fivefold after 24 h) [94] and further to be able to enter the nucleus [95]. In addition, the uptake could be reduced to background levels by GRPR blocking, demonstrating the receptor specificity of the uptake. Despite these promising in vitro results, the in vivo evaluation of the radioligand in PC3 tumor-bearing mice revealed a massive kidney uptake of the agent, exceeding that of the tumor by factor 13 at 2 h p.i., preventing its use in tumor imaging or therapy.

In another, more recent approach, a heterobivalent agent based on a hybrid molecule composed of α_v_β_3_ integrin-specific c(RGDfK) and PSMA-specific iPSMA, being connected by an interpeptidic DOTA-modified cysteine moiety (**24**), was radiolabeled with ^177^Lu and evaluated in vitro regarding its applicability for PCa therapy [96]. However, the radioligand showed lower cell uptakes in U87MG and C6 cells than the corresponding monospecific reference peptides, thus limiting the usefulness of the combined agent and indicating the necessity to improve the molecular design of the compound.

An alternative attempt was made recently, aiming at the bispecific targeting of the GRPR and the VPAC_1_R [66]. The reason for this is that the latter is overexpressed by virtually all PCa primaries and metastases [125,126] and furthermore plays an important role in upregulation of EGFR (epidermal growth factor receptor) and VEGF (vascular endothelial growth factor) [127,128]. Thus, GRPR- and VPAC_1_R-bispecific radiotracers should be able to increase the specificity and sensitivity of prostate carcinoma imaging as well as that of other malignancies expressing these receptor types such as breast cancer. GRPR-specific BBN_7–14_ and VPAC_1_R-specific PACAP-27 (a truncated, 27 amino acid-containing analog of vasoactive intestinal peptide, PACAP = pituitary adenylate-cyclase-activating polypeptide) were linked using a symmetrically branching, NODAGA-comprising interpeptidic linker. Furthermore, linkers of different length and rigidity were introduced to evaluate if a certain distance between both peptide binders or a fixation of molecule geometry yields optimized target interaction, resulting in agents **25**–**28**. The ^68^Ga-labeled agents [^68^Ga]Ga-**25**–[^68^Ga]Ga-**28** were evaluated in vitro in three different prostate carcinoma cell lines (PC3, DU-145, and VCaP) and showed an up to threefold higher uptake into DU-145 cells compared to both monomers, pointing to a synergistic effect of heterodimerization on tumor cell uptake. Additionally, blocking experiments on VCaP cells revealed that both peptide units contribute to tumor cell uptake and thus confirmed the bispecificity of the developed agents. In vivo imaging studies—to show the potential of the radioligands for GRPR- and VPAC_1_R-bispecific imaging— have however to be performed to corroborate the favorable in vitro results.

### 2.4. Heterobivalent Agents Developed for Breast Cancer Imaging or Therapy

**Figure 9:**
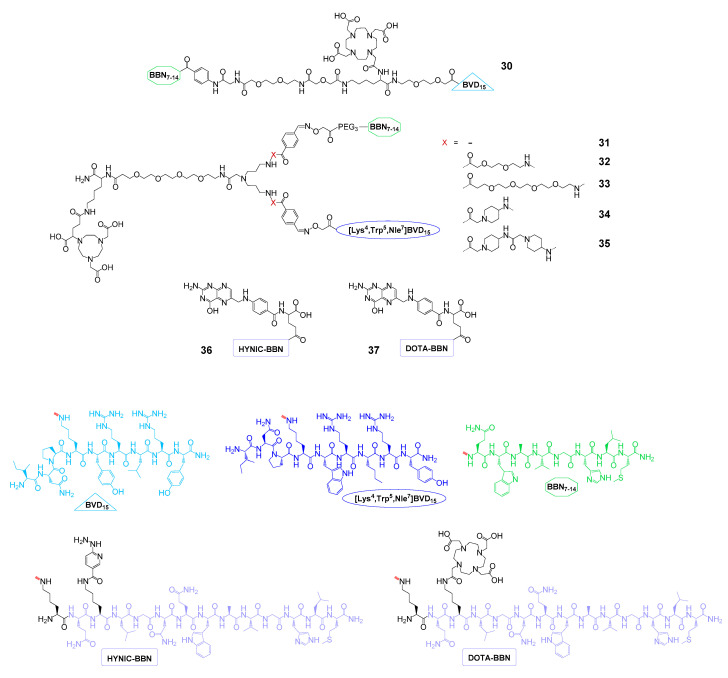
Structures of GRPR- and NPY(Y_1_)R-bispecific and GRPR- and folate receptor-bispecific agents developed for breast cancer imaging or therapy.

Breast cancer overexpresses somatostatin receptors (SSTR), vasoactive intestinal peptide receptors (VIPR), the GRPR, and NPY(Y_1_)R [54]. Out of these, the GRPR and the NPY(Y_1_)R are of particular interest for the development of radiolabeled heterobivalent peptidic agents as they are overexpressed in ≈75% [52] and 66–85% [53] of all breast cancer lesions, respectively, but only to an insignificant amount on healthy breast tissue. Furthermore, most breast cancer lesions (93%) express one or both receptor types [54], enabling a high lesion detection sensitivity in tumor imaging if a bispecific agent can be developed. Thus, different groups aimed to develop GRPR- and NPY(Y_1_)R-bispecific agents (**30**–**35**, Figure 9) for breast cancer imaging.

In a first attempt, **30** was synthesized and evaluated in vitro towards its receptor binding affinities on GRPR-positive T47D and NPY(Y_1_)R-positive MCF7 cells [97]. In these competitive displacement studies, the heterodimer showed considerably decreased affinities towards both receptor types (IC_50(GRPR)_: 18.0 ± 0.7 nM and IC_50(NPY(Y1)R_: 80 ± 11 nM) compared to the monospecific references bombesin (IC_50(GRPR)_: 2.0 ± 0.1 nM) and neuropeptide Y (IC_50(NPY(Y1)R_: 2.6 ± 1.2 nM). No synergistic effect of heterodimerization on tumor cell uptake could be demonstrated. Consequently, the agent was radiolabeled with ^153^Gd and evaluated regarding its stability in human serum [129]. It was demonstrated that the heterobivalent agent did not benefit from heterodimerization in terms of stability against proteolytic degradation although a direct comparison of the values is difficult as the analyses were not carried out at the same time points after the start of the incubation with serum. In comparison, [^153^Gd]Gd-**30** showed only 6.3% intact tracer after 90 min of incubation with human serum, whereas [^153^Gd]Gd-DOTA-BVD_15_, which was tested as the monomeric reference, showed at least 26.3% intact tracer after 75 min. These results prevented further evaluation of **30**.

In another work, heterodimers **31**–**35** were synthesized, radiolabeled with the positron-emitter ^68^Ga, and evaluated in terms of their stability in human serum, in vitro cell uptakes, and in vivo pharmacokinetics [68]. Regarding their stability against proteolytic degradation, all heterodimers showed only a negligible degradation of 1–2% after 90 min incubation in human serum. In in vitro cell uptake studies performed in T47D, MCF-7, BT-474, and MDA-MB-231 cells, the heterodimers showed comparable uptakes as the monospecific GRPR-affine reference [^68^Ga]Ga-DOTA-PESIN (PESIN = PEG_3_-BBN_7–14_), whereas no uptake could be shown for the NPY(Y_1_)R-specific reference [^68^Ga]Ga-DOTA-BVD_15_ due to a low NPY(Y_1_)R density on the used cell lines. Thus, a synergistic effect of peptide heterodimerization could not be proven for the developed agents in the in vitro studies. However, this synergy could be shown in in vivo studies for [^68^Ga]Ga-**33** in T47D tumor-bearing mice where the heterodimer showed absolute higher tumor uptakes and tumor-to-background ratios compared with the respective monospecific, partly scrambled references, demonstrating that both peptide binders of the heterobivalent agent contributed to tumor uptake. Although the proof-of-concept was thus successful, high kidney and liver uptakes were observed in the in vivo experiments, pointing to the necessity to further improve the molecular design of the GRPR- and NPY(Y_1_)R-bispecific agents.

Another concept for highly sensitive breast cancer imaging is the concomitant targeting of the GRPR and FRα, with both being overexpressed on this malignancy [130]. Recently, the synthesis and ^99m^Tc and ^177^Lu radiolabeling and in vitro and in vivo evaluation of two examples of this compound class being able to target both receptors (**36** [98] and **37** [99]) were described. Both agents, only differing in the radiometal chelator system used, were evaluated in vitro regarding their GRPR- and FRα-affinities on T47D tumor cells, both showing affinities to both receptors in the low nanomolar range (IC_50(GRPR)**29**_: 3.2 ± 1.0 nM, IC_50(FRα)**29**_: 6.3 ± 1.5 nM, IC_50(GRPR)**30**_: 4.8 ± 0.9 nM, and IC_50(FRα)**30**_: 9.1 ± 1.5 nM). Furthermore, it was demonstrated in cell uptake and corresponding receptor blocking studies that both parts of the molecule contributed to cell uptake although being mediated to a higher extent by the GRPR. This is a result of the higher GRPR compared to FRα expression that was found on the T47D cells [98]. Both [^99m^Tc]Tc-**36** and [^177^Lu]Lu-**37** were also evaluated in T47D tumor-bearing mice, showing reasonable tumor uptakes of 5.4 ± 1.0% and 6.3 ± 0.7% ID/g at 2 h p.i., respectively, being higher than those of both corresponding monospecific tracers. Only the kidneys showed higher tracer accumulation rates of 11.2 ± 1.4% and 14.6 ± 1.3% ID/g at the same time point whereas all other organs showed significantly lower values. The reasonable tumor retention and fast background clearance of the agents (except kidneys) enables an efficient target visualization at relatively early time points. If it were possible to further decrease kidney uptake, the agents could be of very high potential for breast cancer imaging. An application in tumor therapy seems however not likely due to the relatively low doses applied to the tumor.

### 2.5. Heterobivalent Agents Comprising an α_v_β_3_ Integrin-Binding Peptide for Imaging and Therapy of Different Malignancies

As mentioned before, α_v_β_3_ integrin is overexpressed on a variety of human tumors and during tumor neoangiogenesis and thus lends itself to the development of bispecific agents being able to address tumors with high sensitivity.

**Figure 10:**
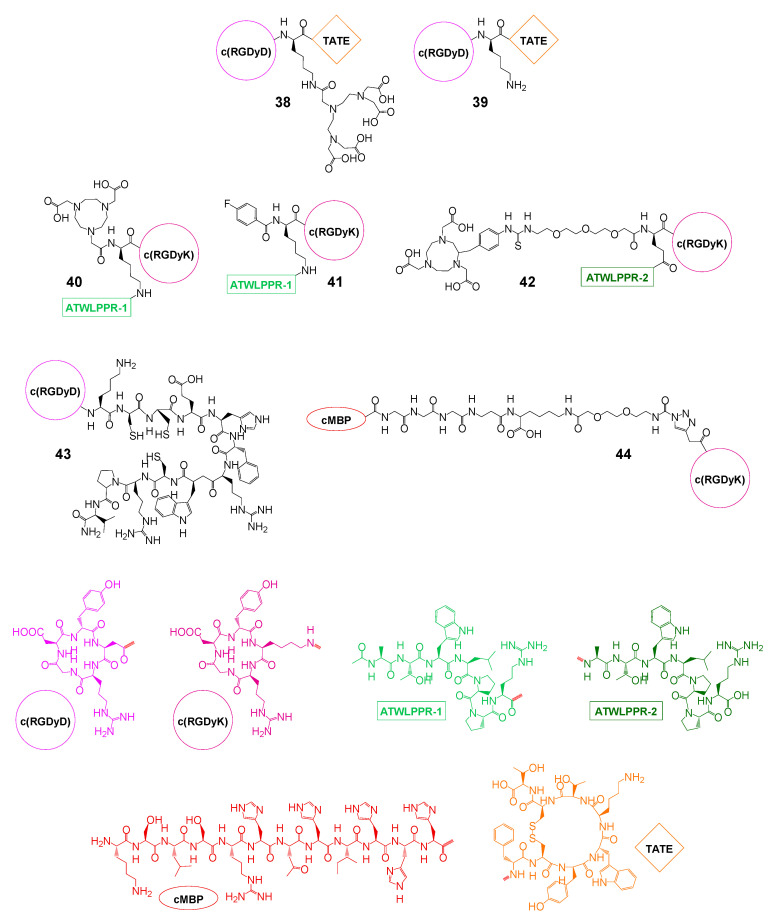
Structures of further bispecific radioligands comprising an α_v_β_3_ integrin-binding peptide developed for imaging or therapy of different malignancies.

Integrin α_v_β_3_-binding peptides were however not only used for tumor targeting but also for the design of peptidic heterodimers intended to exert a pro-apoptotic effect on the target cell by caspase-3 activation, whereas the second peptide enables binding to the target tumor cell. Tyr^3^-octreotate (TATE, truncated analog of the endogenous SSTR ligand somatostatin)-cRGD-heterodimers (**38** and **39**, Figure 10) are an example from this group. The agents were labeled with the SPECT γ-emitter ^111^In (**38**) and ^125^I (**39**) and were in the following evaluated in vitro and in vivo. In a first attempt, [^111^In]In-**38** was evaluated in terms of cytotoxicity compared to the monospecific references [^111^In]In-DTPA-RGD and [^111^In]In-DTPA-TATE in vitro [100]. It was found that [^111^In]In-**38** showed the highest cytotoxicity in SSTR-positive cell lines where the toxicity was directly correlated to the number of SSTRs and mediated by caspase-3 activation. The same group further explored the applicability of the agent in a tumor therapeutic setting in a rat pancreas carcinoma model (CA20948 tumor in rats) in direct comparison to the clinical SPECT standard [^111^In]In-DOTA-TATE [101]. From in vitro cell uptake and receptor blocking studies in the same cell line, it was learned that the SSTR-specific TATE peptide part of the molecule was almost exclusively responsible for tumor uptake whereas the uptake mediated by the RGD peptide part was negligible. This could be confirmed in vivo where SSTR blocking using octreotide resulted in a reduction of the tumor radiotracer uptake to background levels. In direct comparison to [^111^In]In-DOTA-TATE, [^111^In]In-**38** unfortunately showed lower absolute tumor uptakes as well as lower tumor-to-kidney ratios, excluding a use of the latter for therapeutic purposes. In order to reduce the high kidney uptake, the same group developed another, more lipophilic derivative, [^125^I]I-**39**, and tested it in the same preclinical setting [102]. Indeed, the kidney uptake could be reduced, however, resulting in a higher liver uptake of the new agent as compared to [^111^In]In-**38** and still relatively low tumor-to-kidney ratios of 0.42 and 0.96 at 2 h p.i. (depending on which of both ^125^I-labeled products formed was used for the in vivo evaluations). Furthermore, as biodistribution data were not given for all organs of interest and the overall absolute tumor uptakes observed were rather low (1.14 ± 0.21% or 1.91 ± 0.20% ID/g at 2 h p.i. for both possible products), [^125^I]I-**39** also does not seem to be suitable for radiotherapeutic purposes.

The same concept of using heterobivalent peptidic agents comprising an RGD moiety for tumor therapy driven by caspase-3-induced apoptosis induction was also applied in the case of **43**, consisting of c(RGDyD) (cyclo-(Arg-Gly-Asp-*D*Tyr-Asp)) and [Cys^3,4,10^, *D*Phe^7^, Arg^11^]αMSH_3–13_, which binds to the MC1R (melanocortin 1 receptor) for melanoma targeting and uptake [106]. In in vitro MC1R affinity studies on B16-F1 melanoma cells, **43** presented with a high affinity of 2.1 nM (IC_50_ value, no reference compound tested for comparison). In cell uptake studies in the same cell line, the uptake of [^99m^Tc]Tc-**43** could not be blocked using monospecific RGD but only by addition of NDP-MSH, indicating the tumor cell uptake to be solely mediated by the MC1R. In an in vivo B16-F1 melanoma mouse model, the tumor uptake of [^99m^Tc]Tc-**43** was in contrast demonstrated to be driven by both receptor types, the MC1R and α_v_β_3_ integrin, which was verified by blocking experiments using the corresponding monospecific peptides. In biodistribution experiments in the same animal model, the tracer showed a high absolute tumor uptake of 14.8 ± 2.9% ID/g and good tumor-to-background ratios at 2 h p.i. The only exception from this were the kidneys, presenting with an uptake of 67.1 ± 8.8% ID/g at the same time point. This is however less problematic compared to agents labeled with therapeutic radionuclides as the therapeutic effect is in this case to be exerted by the RGD peptide but not by the low amount of introduced ^99m^Tc. Interestingly, [^99m^Tc]Tc-[Cys^3,4,10^, *D*Phe^7^, Arg^11^]αMSH_3–13_, which was evaluated under the same conditions as a monospecific reference compound, exhibited a comparable tumor, but significantly (12.5-fold) lower kidney accumulation at 4 h p.i., which was attributed to the positive charge of the free amino functionality of the interpeptidic lysine residue. Although [^99m^Tc]Tc-**43** was also shown to exert a significant cytotoxicity in a clonogenic survival assay on B16-F1 cells and—apart from the high kidney uptake—a favorable biodistribution profile, the tracer nevertheless requires further molecular optimization to be applicable as a therapeutic agent.

A relatively new but interesting target structure for the design of heterobivalent agents is neuropilin-1 (NRP-1), which interacts with the VEGFR as a co-receptor and is correlated with the vascularization, progression, and metastasis of different cancers such as glioma, prostate, breast, colon, and lung cancers [131]. A peptide being able to target this receptor, ATWLPPR (sequence Ala-Thr-Trp-Leu-Pro-Pro-Arg), was identified recently, showing, however—in ^99m^Tc-labeled, monospecific form—low tumor uptakes in in vivo biodistribution experiments due to fast excretion [132]. To circumvent this issue, attempts were made to improve tumor uptakes and further enhance target specificity and imaging sensitivity of ATWLPPR by its heterodimerization with integrin α_v_β_3_-specific c(RGDyK). For this purpose, ATWLPPR was conjugated via its *C*-terminus (**40** and **41**) or its *N*-terminus (**42**) to a lysine or glutamic acid interpeptidic core structure, also carrying, besides the RGD peptide, functionalities for the introduction of a radiolabel. In **40**, a NOTA chelate was introduced into the molecule enabling radiolabeling using the [^18^F]AlF-labeling strategy [103]. Furthermore, **40** was evaluated in vitro in competitive displacement assays in comparison to the monomeric references on dual α_v_β_3_- and NRP-1-positive U87MG cells, showing comparable IC_50_ affinity values of heterodimer and monomers in the two-digit nM range to both receptor types and thus a specific binding to both target structures. The bi-specificity of [^18^F]F-**40** could also be confirmed by in vivo PET imaging studies in U87MG tumor-bearing mice, showing only a partial blocking of tumor uptake using either c(RGDyK) or ATWLPPR, but the combined application of both blocking agents resulted in a reduction of tumor uptakes to background levels. Furthermore, the heterobivalent peptide reached higher absolute tumor uptakes (≈5.0% ID/g at 1 h p.i.) than both monospecific references [^18^F]AlF-NOTA-ATWLPPR (≈2.2% ID/g at 1 h p.i.) and [^18^F]AlF-NOTA-c(RGDyK) (≈2.8% ID/g at 1 h p.i.). Unfortunately, the tracer also showed a significant background accumulation in kidneys (≈2.8% ID/g at 1 h p.i.), liver (≈4.1% ID/g at 1 h p.i.), intestines (≈1.8% ID/g at 1 h p.i.), and bone (≈3.3% ID/g at 1 h p.i.), pointing on the one hand to a liberation of the ^18^F-fluoride and on the other hand to the need for improvement of the pharmacokinetic profile of the agent. The same group in the following tested the influence of using another labeling strategy of the heterobivalent core structure on in vivo pharmacokinetics. Instead of [^18^F]AlF-labeling, [^18^F]SFB was used for ^18^F-introduction, resulting in comparable in vitro properties of the compound, however—as expected—a considerably less favorable in vivo pharmacokinetic profile of [^18^F]**41** compared to [^18^F]F-**40**, with lower absolute tumor uptake (≈1.5% ID/g at 1 h p.i.) and decreased tumor-to-background ratios [104]. Another attempt to enhance the in vivo pharmacokinetics of the bispecific ligands by conjugating the NRP-1-specific peptide via its *N*-terminus instead of the *C*-terminus and using a NODAGA instead of a NOTA chelator for [^18^F]AlF-labeling yielding **42** was also unable to improve the properties of the agent, still showing a considerable background accumulation in the gastrointestinal tract although at least a higher stability of ^18^F-fluoride incorporation was able to be achieved, being reflected in a lower ^18^F bone accumulation [105].

Finally, a combination of α_v_β_3_-binding c(RGDyK) and a peptide specifically targeting the mesenchymal–epithelial transition factor (c-Met) was reported. The c-Met is overexpressed in a variety of tumors and is associated with invasive cancer growth, increased metastatic potential, proliferation and vascularity [133]. Thus, the concomitant targeting of both c-Met and α_v_β_3_ could result in a higher target visualization sensitivity and specificity compared with using one peptide alone. However, only one example of this group of bispecific agents has been described thus far (**44**), connecting both peptidic binders via a relatively long hydrophilic linker structure element [107]. In vitro competitive displacement studies on both α_v_β_3_- and c-Met-positive U87MG cells revealed low binding affinities of the heterodimer to both receptor types (IC_50(αvβ3)_: 3.42 µM and IC_50(c-Met)_: 3.84 µM) and also of the tested monospecific reference peptides c(RGDyk) (IC_50(αvβ3)_: 2.38 µM) and cMBP-GGG-SC (IC_50(c-Met)_: 1.53 µM) to their respective targets. Consequently, the in vivo biodistribution data of [^125^I]I-**44** in U87MG tumor-bearing mice demonstrated a high background accumulation of the tracer in kidneys (T/K 0.22), stomach (T/S 0.47), pancreas (T/P 0.28), and other organs at 1 h p.i., as well as a high thyroid uptake of the tracer being obvious in in vivo SPECT images, indicating a low in vivo stability of the radioligand, impeding its application in tumor imaging.

## 3. Outlook

Radiolabeled heterobivalent peptidic agents have the potential to considerably improve tumor imaging in the future, not only by delineating the target with higher specificity and efficiency but also by increasing target visualization sensitivity due to their ability to bind to different receptors in synergy. This solves the ubiquitous problem of heterogeneous receptor expression between different tumor lesions, for instance occurring during tumor progression or metastasis, often resulting in ineffective detection of all lesions using monospecific agents. The value of such radiolabeled heterobivalent peptidic agents for tumor imaging has been demonstrated in the clinical context recently and the interest in this field of research is steadily growing.

The findings obtained thus far however also reveal the importance to systematically determine the receptor expression profiles and receptor densities on the target malignancies in order to identify those receptor types that lend themselves most appropriate for concomitant targeting.

Aside from the discussed heterodimers developed thus far, other peptide combinations might be promising and will most probably yield new radiotracers with high potential for tumor imaging and further descriptions of some of the already developed agents in a clinical context within the next few years.

## Figures and Tables

**Table 1:** Radiolabeled heterobivalent peptides developed for tumor imaging or therapy thus far.

Target Receptors	Compound Number	Radionuclide Used	Intended Application	In Vitro Affinity Data	Model Used for In Vivo Evaluation	Reference
GRPR and PSMA	**1**	^64^Cu	PET imaging	IC_50(GRPR)_: 11.1 ± 0.5 nM, IC_50(PSMA)_: 1.2 ± 1.4 nM	PC3/AR42J and LNCaP mice	[74]
	**2**	^68^Ga	PET imaging	IC_50(GRPR)_: 9.0 ± 1.8 nM, IC_50(PSMA)_: 25.0 ± 5.4 nM	PC3/AR42J and LNCaP mice	[75]
	**3**–**5**	^68^Ga	PET imaging	**3**: IC_50(GRPR)_: 7.3 nM, IC_50(PSMA)_: 17.4 nM **4**: IC_50(GRPR)_: 4.4 nM, IC_50(PSMA)_: 25.2 nM **5**: IC_50(GRPR)_: 7.1 nM, IC_50(PSMA)_: 42.4 nM	PC3 and LNCaP mice	[76]
	**6**	^68^Ga and ^177^Lu	PET imaging and therapy	[^68^Ga]Ga-**6**: K_d(GRPR)_: 43.7 ± 3.8 nM, K_d(PSMA)_: 4.4 ± 2.3 nM [^177^Lu]Lu-**6**: IC_50(GRPR)_: 3.5 ± 0.4 nM, IC_50(PSMA)_: 5.6 ± 1.5 nM	PC3 and LNCaP mice	[77,78]
	**7**	^68^Ga and ^111^In	PET and SPECT imaging	IC_50(GRPR)_: 4 ± 1 nM, IC_50(PSMA)_: 824 ± 230 nM	PC3-PIP mice	[79]
	**8**–**10**	^125^I	SPECT imaging and therapy	**8**: IC_50(GRPR)_: 6 ± 2 nM, IC_50(PSMA)_: 80 ± 7 nM **9**: IC_50(GRPR)_: 13 ± 3 nM, IC_50(PSMA)_: 98 ± 13 nM **10**: IC_50(GRPR)_: 20 ± 2 nM, IC_50(PSMA)_: 100 ± 10 nM	PC3 and LNCaP mice	[80]
GRPR and α_v_β_3_	**11**	^18^F	PET imaging	IC_50(GRPR)_: 32.0 ± 1.9 nM, IC_50(αvβ3)_: 282 ± 34 nM	PC3 mice	[65]
	**12**	^18^F	PET imaging	IC_50(GRPR)_: 73.3 ± 1.6 nM, IC_50(αvβ3)_: 13.8 ± 1.8 nM	PC3 mice	[64]
	**13**	^18^F	PET imaging	IC_50(GRPR)_: 167 ± 1 nM, IC_50(αvβ3)_: 553 ± 1 nM	PC3 mice	[81]
	**14**	^68^Ga and ^64^Cu	PET imaging	IC_50(GRPR)_: 92.8 ± 3.5 nM, IC_50(αvβ3)_: 16.2 ± 2.8 nM	T47D and MDA-MB-435 mice	[82]
		^68^Ga	PET imaging	IC_50(GRPR)_: 55.9 ± 4.2 nM, IC_50(αvβ3)_: 22.6 ± 6.7 nM	PC3 mice	[83]
		^68^Ga	PET imaging	-	PCa patients	[84]
	**15**	^64^Cu	PET imaging	IC_50(GRPR)_: 4.0 ± 0.4 nM, IC_50(αvβ3)_: no affinity	PC3 mice	[85]
	**16**	^64^Cu	PET imaging	IC_50(GRPR)_: 85.8 ± 2.1 nM, IC_50(αvβ3)_: 21.6 ± 2.2 nM	PC3 mice	[86]
		^177^Lu	PET imaging	-	PC3 mice	[87]
	**17**	^64^Cu	PET imaging	IC_50(GRPR)_: 3.1 ± 0.3 nM, IC_50(αvβ3)_: 518 ± 38 nM	PC3 mice	[88]
	**18**	^86^Y and ^90^Y	PET imaging and therapy	IC_50(GRPR)_: 5.7 ± 0 nM, IC_50(αvβ3)_: 346 ± 5 nM	PC3 mice	[89]
		^111^In and ^177^Lu	SPECT imaging and therapy	^nat^In-IC_50(GRPR)_: 5.4 ± 1.4 nM, ^nat^In-IC_50(αvβ3)_: 372 ± 23 nM; ^nat^Lu-IC_50(GRPR)_: 5.8 ± 3.2 nM, ^nat^Lu-IC_50(αvβ3)_: 346 ± 53 nM	PC3 mice	[90]
	**19**	^64^Cu	PET imaging	IC_50(GRPR)_: 24.3 ± 10.9 nM, IC_50(αvβ3)_: 165.3 ± 105.4 nM	PC3 mice	[91]
	**20**	^64^Cu	PET imaging	IC_50(GRPR)_: 100.4 ± 73.2 nM, IC_50(αvβ3)_: 101.2 ± 57.4 nM	PC3 mice	[91]
	**21**	^99m^Tc	SPECT imaging	IC_50(GRPR)_: 104.7 ± 5.8 nM, IC_50(αvβ3)_: 18.8 ± 3.7 nM	LLC mice	[92]
	**22**	^99m^Tc and ^122^Re	SPECT imaging and therapy	IC_50(GRPR)_: 63.3 ± 2.9 nM, IC_50(αvβ3)_: 13.4 ± 2.5 nM	PC3 mice	[93]
GRPR and TAT	**23**	^99m^Tc	SPECT imaging and therapy	cell uptake studies, no affinities	PC3 mice	[94,95]
PSMA and α_v_β_3_	**24**	^177^Lu	therapy	IC_50(PSMA)_: 1.69 nM, IC_50(αvβ3)_: 1.05 nM	-	[96]
GRPR and VPAC_1_R	**25–29**	^68^Ga	PET imaging	cell uptake studies, no affinities	-	[66]
GRPR and NPY(Y_1_)R	**30**	^153^Gd	-	IC_50(GRPR)_: 18.0 ± 0.7 nM, IC_50(NPY(Y1)R)_: 80 ± 11 nM	-	[97]
	**31**–**35**	^68^Ga	PET imaging	-	T47D mice	[68]
GRPR and FRα	**36**	^99m^Tc	SPECT imaging	IC_50(GRPR)_: 3.2 ± 1.0 nM, IC_50(FRα)_: 6.3 ± 1.5 nM	T47D mice	[98]
	**37**	^177^Lu	SPECT imaging and therapy	IC_50(GRPR)_: 4.8 ± 0.9 nM, IC_50(FRα)_: 9.1 ± 1.5 nM	T47D mice	[99]
α_v_β_3_ and SSTR	**38**	^111^In	(SPECT imaging) therapy	IC_50(SSTR)_: 94 nM, IC_50(_α_v_β_3)_: n.d.	CA20948 rats	[100,101,102]
	**39**	^125^I	therapy	IC_50(SSTR)_: 14 nM, IC_50(_α_v_β_3)_: n.d.	CA20948 rats	[102]
α_v_β_3_ and NRP-1	**40**	^18^F	PET imaging	IC_50(NRP-1)_: 60.1 ± 6.5 nM, IC_50(_α_v_β_3)_: 43.8 ± 4.8	U87MG mice	[103]
	**41**	^18^F	PET imaging	IC_50(NRP-1)_: 23.7 nM, IC_50(_α_v_β_3)_: 21.7 nM	U87MG mice	[104]
	**42**	^18^F	PET imaging	IC_50(U87MG)_: 44.2 nM	U87MG mice	[105]
α_v_β_3_ and MC1R	**43**	^99m^Tc	SPECT imaging and therapy	IC_50(B16-F1)_: 2.1 nM	B16-F1 mice	[106]
α_v_β_3_ and c-Met	**44**	^125^I	SPECT imaging	IC_50(c-Met)_: 3.84 µM, IC_50(_α_v_β_3)_: 3.42 µM	U87MG mice	[107]

**Table 2:** Decay characteristics of radionuclides used for heterobivalent peptide labeling [108,109,110].

Radionuclide	Decay Mode	Half-Life	Application	Mean β-/γ-Energy
^18^F	β^+^ (100%)	109.77 min	PET imaging	249.8 keV (β^+^)
^64^Cu	β^+^ (17.6%) β^−^ (38.5%)	12.70 h	PET imaging	278.2 keV (β^+^)
^68^Ga	β^+^ (89.1%)	67.71 min	PET imaging	836.0 keV (β^+^)
^86^Y	β^+^ (31.9%)	14.74 h	PET imaging	535 keV (β^+^)
^90^Y	β^−^ (100%)	2.67 days	therapy	933 keV (β^−^)
^99m^Tc	γ (100%)	6.01 h	SPECT imaging	140.51 keV (γ)
^111^In	EC (100%)	2.80 days	SPECT imaging	171.28 keV (γ) 245.35 keV (γ)
^125^I	EC (100%)	60 days	therapy	35.5 keV (γ)
^177^Lu	β^−^ (100%)	6.71 days	therapy	133 keV (β^−^)

β^+^ = positron emission. β^−^ = electron emission. EC = electron capture.
